# Colorectal cancer-infiltrating T lymphocytes display a distinct chemokine receptor expression profile

**DOI:** 10.1186/s40001-017-0283-8

**Published:** 2017-10-11

**Authors:** Ann-Britt Löfroos, Mohammad Kadivar, Sabina Resic Lindehammer, Jan Marsal

**Affiliations:** 1 Department of Clinical Sciences, Lund University, Lund, Sweden; 2 Immunology Section, Lund University, Lund, Sweden; 3 Department of Gastroenterology, Skane University Hospital, 22185 Lund, Sweden

**Keywords:** Chemokine receptor, T lymphocyte, Colorectal cancer, Mucosa, Homing

## Abstract

**Background:**

T lymphocytes exert important homeostatic functions in the healthy intestinal mucosa, whereas in case of colorectal cancer (CRC), infiltration of T lymphocytes into the tumor is crucial for an effective anti-tumor immune response. In both situations, the recruitment mechanisms of T lymphocytes into the tissues are essential for the immunological functions deciding the outcome. The recruitment of T lymphocytes is largely dependent on their expression of various chemokine receptors. The aim of this study was to identify potential chemokine receptors involved in the recruitment of T lymphocytes to normal human colonic mucosa and to CRC tissue, respectively, by examining the expression of 16 different chemokine receptors on T lymphocytes isolated from these tissues.

**Methods:**

Tissues were collected from patients undergoing bowel resection for CRC. Lymphocytes were isolated through enzymatic tissue degradation of CRC tissue and nearby located unaffected mucosa, respectively. The expression of a broad panel of chemokine receptors on the freshly isolated T lymphocytes was examined by flow cytometry.

**Results:**

In the normal colonic mucosa, the frequencies of cells expressing CCR2, CCR4, CXCR3, and CXCR6 differed significantly between CD4^+^ and CD8^+^ T lymphocytes, suggesting that the molecular mechanisms mediating T lymphocyte recruitment to the gut differ between CD4^+^ and CD8^+^ T lymphocytes. In CRC, the frequencies of cells expressing CCR2 and CXCR5 were significantly lower in both the CD4^+^ and CD8^+^ T lymphocyte populations compared to unaffected colonic mucosa, and the frequency of CCR9^+^ cytotoxic T lymphocytes was significantly decreased in CRC tissue.

**Conclusions:**

With regard to the normal gut mucosa, the results suggest that the molecular mechanisms mediating T lymphocyte recruitment differ between CD4^+^ and CD8^+^ T lymphocytes, which are important for understanding gut homeostasis. Importantly, T lymphocytes from CRC compared to normal colonic tissue displayed a distinct chemokine receptor expression profile, suggesting that mechanisms for recruitment of T lymphocytes to CRC tissue are skewed compared to normal colonic mucosa. Understanding these mechanisms could help in developing new strategies in cancer immunotherapy and to optimize already available alternatives such as immune checkpoint inhibitors.

## Background

Colorectal cancer (CRC) is the third most common type of cancer in adults and the second leading cause of cancer death in Western countries [1]. Typically, the patient raises an adaptive immune response towards the tumor, giving rise to tumor-infiltrating T lymphocytes (TIL) [2, 3]. The lymphocytes are recruited from the blood circulation to the tumor site and infiltrate the tumor mass; a sequential process directed by adhesion molecules, chemokines, and chemokine receptors.

Chemokines constitute a large family (48 known human members) of small (7–10 kDa) peptides that are classified according to the position of their conserved cysteine residues into four groups: CXC, CC, C, and CX3C [4, 5]. Based on their function, they are referred to as homeostatic, inflammatory, or both [6]. Chemokines mediate their effects by binding to 7-transmembrane G-protein coupled receptors expressed on target cells [4]. There are 18 identified human chemokine receptors (and 5 additional atypical receptors), grouped according to the type of ligand they bind, and have been named CXCR1 through 6, CCR1 through 10, CX3CR1, and XCR1 [7].

Chemokines are mainly known for their ability to direct leukocyte recruitment and migration under both homeostatic and inflammatory conditions [6, 8, 9]. Homeostatic chemokines (constitutively expressed) are important for several physiological processes such as embryogenesis, hematopoiesis, and lymphocyte trafficking [8, 10], whereas the expression of inflammatory chemokines is induced upon pathological events, such as inflammation and cancer growth [8, 11]. Furthermore, in cancer biology, chemokines play a role in a number of additional processes, such as tumor cell growth/survival, metastatic spreading, and angiogenesis [11–13].

The colonic mucosa is lined by a single-cell layer of columnar epithelium sitting on a laminin/collagenous basement membrane. Underlying this is a layer of loose connective tissue called *lamina propria* (LP), which in turn is demarcated by a thin smooth muscular layer (*muscularis mucosae*). The formation of CRC is thought to start through inappropriate epithelial proliferative and antiapoptotic activity leading to formation of adenomas, which evolve into pre-invasive carcinoma in situ [14]. Pre-invasive CRCs eventually acquire the ability to invade through the submucosa and *muscularis propria*, and finally to metastasize [14]. The progression from normal epithelium through adenoma to colorectal carcinoma is characterized and driven by multiple accumulated mutations of cancer genes [15].

In normal colonic mucosa, effector lymphocytes that localize to the epithelial compartment (intraepithelial lymphocytes; IEL) are primarily CD8^+^ T lymphocytes. Interestingly, IEL have been implicated in the control of intestinal epithelial cell growth/turnover and in sensing and eliminating cancerous or injured epithelial cells [16, 17]. In contrast, the LP contains mainly CD4^+^ T lymphocytes (*lamina propria* lymphocytes; LPL) at an approximate CD4/CD8 ratio of 2:1 at homeostatic conditions and is thought to be important in maintaining a tolerogenic environment [18]. Colonic T lymphocyte homing is less studied than homing to the small intestine, but likely requires either integrin α4β7 or α4β1 [19–21]. In addition, CCR2, CCR5, CCR6, CXCR3, CXCR4, and CXCR6 expression has been observed on proportions of inflamed and non-inflamed colonic mucosal T lymphocytes in humans [22–26].

Cancer tissue is typically infiltrated by effector T lymphocytes and the presence of these TILs has been correlated with improved clinical outcome in different human cancers, including CRCs [2, 3, 27, 28]. In a study by Galon et al., patients with high frequencies of infiltrating CD3^+^ T lymphocytes within their colorectal tumors had a better 5-year survival rate (73%) than those with low numbers of CD3^+^ T lymphocytes (30%) [29]. Furthermore, it has been shown that both CD4^+^ and CD8^+^ effector T lymphocytes may have anti-tumor properties and independently correlate with improved outcome [30–32]. Thus, TILs and their ability to localize to CRCs constitute attractive targets for cancer immunotherapy. Indeed, new anti-cancer therapies, such as ipilimumab which is a blocking antibody to cytotoxic T lymphocyte-associated protein-4 (CTLA-4), are associated with increased infiltration of T lymphocytes into the tumor tissue and significantly increased survival rates [33]. Despite the fact that these studies recognize the protective effect of infiltrating T lymphocytes against cancer and that the initial studies regarding TILs were published almost 30 years ago, the molecular mechanisms whereby TILs are recruited into tumors remain elusive [34, 35].

A number of chemokines, such as CCL2–4, CXCL1, CXCL5, and CXCL8–10, have been shown to be present at elevated levels in the CRC microenvironment compared to normal tissues [36, 37]. However, it remains unclear which chemokine receptors T lymphocytes use to infiltrate into the tumor mass. The previous studies have presented data for a few of the known chemokine receptors regarding the expression on TILs in CRC (i.e., CCR2, CCR4–7, and CXCR3) [38–43]. The results of a recently published study showed that CXCR3 was expressed on significantly fewer CD4^+^ and CD8^+^ T lymphocytes in tumor tissue compared to the unaffected tissue [44]. In contrast, there was a higher frequency of CD4^+^ T lymphocytes expressing CCR4 in the tumor tissue [44].

The aim of the current study was to identify potential chemokine receptors involved in T lymphocyte recruitment to human normal colonic mucosa and to CRC tissue. We examined the expression of 16 chemokine receptors on CD4^+^ and CD8^+^ T lymphocytes in normal colonic mucosa as compared with CRC tissue. The results showed that CD4^+^ and CD8^+^ T lymphocytes in unaffected colonic mucosa have distinct chemokine receptor profiles, and furthermore that T lymphocytes from unaffected colonic tissue as compared with CRC tissue differ in their chemokine receptor profile.

## Methods

### Tissue samples

Tissue specimens were obtained from six patients undergoing bowel resection for CRC. The tumors were of primary and invasive type. The age of the patients ranged from 45 to 78 years, with an average of 62.5 years. During the colectomy procedure, a piece of tumor tissue and that of unaffected mucosa at least 5 cm away from the tumor were collected and immediately transported in 4 °C Hank’s solution to the laboratory facilities. The size of the tumor tissue and unaffected tissue specimens was 2–3 and 10–15 cm^2^, respectively. The study was approved by the regional research ethics committee at Lund University, Sweden (LU 75-03, LU 132-98). Informed written consent was obtained from the patients and the study conformed to the ethical guidelines of the Declaration of Helsinki.

### Isolation of lymphocytes

Lymphocytes were obtained from surgical samples of unaffected human colonic mucosa and carcinoma tissue by a previously described method with minor modifications [45]. Briefly, the mucosa was dissected free of underlying adipose tissue and musculature along the fibrous connective tissue layer. All remaining fibrous tissue strands were carefully removed and specimens were cut into 1 cm^2^ tissue pieces. Epithelial cells were removed by EDTA (0.5 M) treatment at 37 °C for 3 × 30 min. The remaining tissue was incubated in RPMI-10 containing collagenase type VIII (100 U/mL, Sigma-Aldrich) and CaCl_2_ (0.5 M), for 1 h. The resulting cell suspension was spun down and further purified using a 40/70% Percoll gradient (Percoll™, GE Healthcare). After centrifugation, the lymphocytes were aspirated at the 40/70 interface. The cells were washed with RPMI-10 medium and the total numbers of viable lymphocytes were enumerated by light microscopy and trypan blue dye exclusion.

### Flow cytometry analysis of intestinal lymphocyte populations

Purified lymphocytes from tissue samples of unaffected mucosa and carcinoma tissue were incubated with 10 µg/mL mouse or rabbit anti-human chemokine receptor antibody for 40 min (after a 20 min blocking step with 3% normal human serum and 3% BSA). Sixteen human chemokine receptors were stained for primary mouse or rabbit antibodies (Table 1) obtained from Abcam, Becton–Dickinson (BD), R&D Systems (R&D S), Sigma-Aldrich (S-A), or kindly provided by Millenium Pharmaceuticals (MP). The secondary antibodies, biotinylated goat anti-mouse or goat anti-rabbit (1:200), were applied and incubated for 40 min. Finally, a reagent mix consisting of FITC-, and PE-conjugated monoclonal human-anti-mouse CD4 (1:50) and CD8 (1:50) antibodies, streptavidin-APC (1:800) (Becton–Dickinson), and a fluorescent DNA-binding probe, 7-amino-actinomycin D (7AAD, 1:200; for identification of non-viable cells) was added to the cells. All flow cytometry analyses were performed with FACSCalibur flow cytometer using the CellQuest™ software to acquire and analyze the FACS plots (Becton–Dickinson).

**Table 1:** Broad panel of monoclonal or polyclonal antibodies to human chemokine receptors together with isotype matched negative controls was used in the current study

Receptor	Clone	Isotype	Source
CCR1	2D4	IgG1	MP
CCR2	1D9	IgG2a	MP
CCR3	7B11	IgG2a	MP
CCR4	1G1	IgG1	MP
CCR5	2D7	IgG1	MP
CCR6	11A9	IgG1	MP
CCR8	ab8019	Polycl. rabbit	Abcam
CCR9	gpr96, 1.3	IgG1	MP
CCR10	IB5; 5H3	IgG2a; IgG1	MP
CXCR1	5A12	IgG2b	MP
CXCR2	6C6	IgG1	BD
CXCR3	1C6	IgG1	BD
CXCR4	12G5	IgG2a	MP
CXCR5	BLR-1	IgG2b	R&D S
CXCR6	7F3-8-1	IgG1	MP
CX3CR1	ab8020	Polycl. rabbit	Abcam
Neg. control	–	IgG1/2a/2b	BD
Neg. control	–	Rabbit serum	S-A

### Statistical analysis and data presentation

Statistical analyses were performed using Prism Software version 6.0 (GraphPad Software) and reported values represent median values with interquartile range (IQR). *p* < 0.05 were considered statistically significant.

## Results

### Colorectal carcinoma tissues were infiltrated by T lymphocytes

**Fig. 1:**
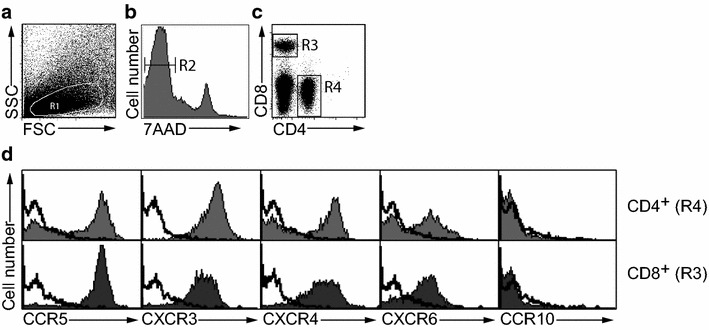
Flow cytometry gating strategy for chemokine receptor expression on T lymphocytes. **a**–**c** Representative plots show the gating strategy to define viable CD4^+^ and CD8^+^ T lymphocytes. **d** Representative flow cytometry histograms of the chemokine receptors CCR5, CXCR3, CXCR4, CXCR6, and CCR10 on CD4^+^ and CD8^+^ lymphocytes isolated from unaffected mucosa. Shaded histograms indicate the specific antibody and open histograms the isotype control

To examine the expression profile of chemokine receptors on T lymphocytes recruited to unaffected colonic mucosal tissue and colorectal carcinoma (CRC) tissue, we isolated lymphocytes from human CRC specimens and unaffected *lamina propria*, for flow cytometry analysis. In accordance with the previous studies showing that T lymphocytes infiltrate into tumor tissue (2, 29), our results showed that the mean number of viable lymphocytes, based on trypan blue exclusion, was higher in carcinoma tissues (5.2 × 10^6^ cells/cm^2^) compared with unaffected tissues (1.2 × 10^6^ cells/cm^2^). Viable (i.e., 7AAD negative) CD4^+^ and CD8^+^ lymphocyte populations were gated, as shown in Fig. 1a–c, for further analysis of the expression of 16 different human chemokine receptors. Figure 1d shows representative flow cytometry histograms of the chemokine receptors CCR5, CCR10, CXCR3, CXCR4, and CXCR6 on CD4^+^ and CD8^+^ lymphocytes isolated from unaffected mucosa.

### The chemokine receptor expression profiles differed between CD4^+^ and CD8^+^ mucosal T lymphocytes

**Fig. 2:**
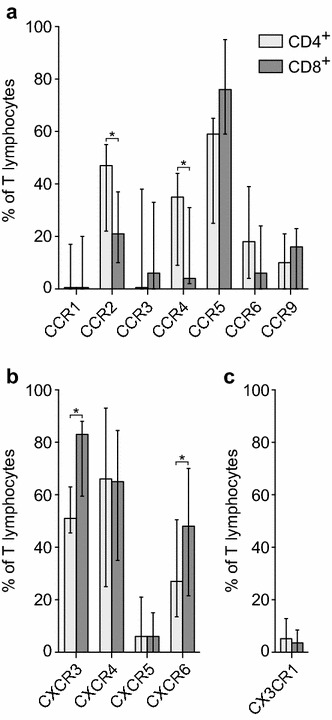
Chemokine receptor expression profiles differed between mucosal CD4^+^ and CD8^+^ T lymphocytes. The bars show median CC (**a**), CXC (**b**), and CX3C (**c**) chemokine receptor expression frequencies on human CD4^+^ (light gray bars) versus CD8^+^ (dark gray bars) viable lamina propria T lymphocytes isolated from normal colonic mucosa, as analyzed by flow cytometry. The vertical lines represent the interquartile range (IQR). Wilcoxon test; *p < 0.05

To examine the physiological levels of chemokine receptor expression on human colonic mucosal T lymphocytes, we first analyzed lymphocytes isolated from unaffected mucosal *lamina propria*. The receptors CCR5, CXCR3, and CXCR4 were expressed by high percentages of CD4^+^ T lymphocytes with median expression frequencies of 59.0% (IQR 30.0–62.5), 51.0% (IQR 45.5–63.0), and 66.0% (IQR 25.0–93.0), respectively (Fig. 2a–c). CCR2 (47.0% IQR 27.0–52.5), CCR4 (35.0% IQR 15.5–39.5), CCR6 (18.0% IQR 10.0–31.5), and CXCR6 (27.0% IQR 13.5–50.5) showed somewhat lower expression frequencies, whereas CCR1 (0.0% IQR 0.0–11.5), CCR3 (0.0% IQR 0.0–22.5), CCR9 (10.0% IQR 3.0–18.0), CXCR5 (6.0% IQR 0.0–21.0), and CX_3_CR1 (0.0% IQR 0.0–13.0) were expressed by small proportions of CD4^+^ T lymphocytes (Fig. 2a–c).

Similar to the CD4^+^ T lymphocytes, CCR5 (76.0% IQR 60.3–93.2), CXCR3 (83.0% IQR 59.5–88.0), and CXCR4 (65.0% IQR 35.0–84.0) were expressed on the majority of CD8^+^ T lymphocytes. In addition, CCR2 (21.0% IQR 15.5–35.5) and CXCR6 (48.0% IQR 21.5–70.0) showed rather high expression frequencies on the CD8^+^ T lymphocytes. In contrast, CCR1 (0.0% IQR 0.0–10.0), CCR3 (6.0% IQR 0.0–26.5), CCR4 (4.0% IQR 2.0–21.0), CCR6 (6.0% IQR 2.0–20.0), CCR9 (16.0% IQR 7.5–21.5), CXCR5 (6.0% IQR 0.0–15.0), and CX_3_CR1 (0.0% IQR 0.0–9.0) were expressed by lower percentages of the CD8^+^ T lymphocytes (Fig. 2a–c). CCR8, CCR10, CXCR1, and CXCR2 were not expressed by neither the CD4^+^ nor the CD8^+^ T lymphocyte populations.

When comparing the expression levels of chemokine receptors on CD4^+^ versus CD8^+^ T lymphocytes from unaffected *lamina propria*, CCR2 and CCR4 showed significantly higher expression frequencies on CD4^+^ T lymphocytes than on CD8^+^ T lymphocytes (Fig. 2a). In contrast, CXCR3 and CXCR6 were more frequently expressed on CD8^+^ than on CD4^+^ lymphocytes (Fig. 2b). CCR5 showed a tendency towards higher expression on CD8^+^ lymphocytes, but this difference was not statistically significant. The remaining chemokine receptors were expressed at similar levels by CD4^+^ and CD8^+^ T lymphocyte populations (Fig. 2a–c).

### T lymphocytes from colorectal carcinoma tissue showed a distinct chemokine receptor expression profile compared to unaffected tissue

**Fig. 3:**
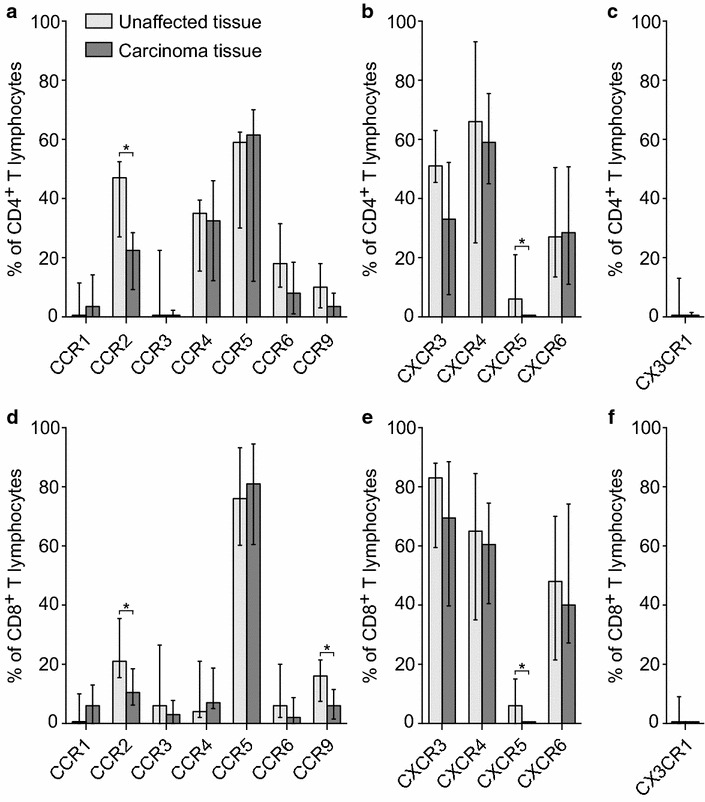
T lymphocytes from colorectal carcinoma tissue showed a distinct chemokine receptor expression profile compared to unaffected tissue. The bars show median chemokine receptor expression frequencies on human CD4^+^ (**a**–**c**) and CD8^+^ (**d**–**f**) viable T lymphocytes isolated from unaffected colonic mucosa (light gray bars) versus colonic carcinoma tissue (dark gray bars), as analyzed by flow cytometry. The vertical lines represent the interquartile range (IQR). Wilcoxon test; *p < 0.05

Next, we compared the expression frequencies of chemokine receptors on CD4^+^ (Fig. 3a–c) and CD8^+^ lymphocytes (Fig. 3d–f) in unaffected mucosa versus carcinoma tissue. The overall average of the chemokine receptor expression frequencies showed that the T lymphocytes isolated from carcinoma tissues expressed lower levels of chemokine receptors (20.1 ± 5.6%) than those isolated from unaffected mucosa (26.9 ± 5.8%) (Wilcoxon test; *p* < 0.01) in CD4^+^ lymphocytes. The same was true for CD8^+^ lymphocytes demonstrating numbers of 24.3 ± 8.4 and 28.8 ± 8.1%, respectively (Wilcoxon test; *p* < 0.01).

**Fig. 4:**
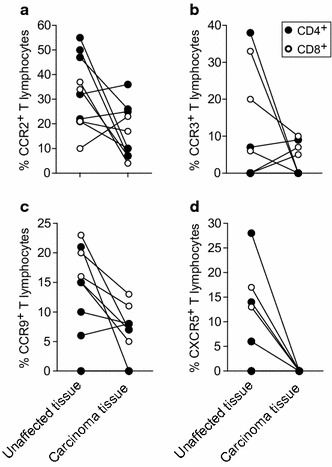
Expression frequencies of CCR2, CCR3, CCR9, and CXCR5 on T lymphocytes were lower in CRC tissue as compared with normal tissue. Percentages of CCR2^+^ (**a**), CCR3^+^ (**b**), CCR9^+^ (**c**), and CXCR5^+^ (**d**) cells among CD4^+^ (closed circles) and CD8^+^ (open circles) T lymphocytes isolated from colonic carcinoma tissue compared with unaffected colonic mucosa. Data points from the same patient for CD4^+^ or CD8^+^ T lymphocytes, respectively, are connected

The percentages of CCR2^+^ cells in both subsets of CD4^+^ and CD8^+^ T lymphocytes were significantly lower in carcinoma tissue as compared with unaffected mucosa (Figs. 3a, b, 4a). Moreover, the percentages of CCR9^+^CD8^+^ and CCR9^+^CD4^+^ lymphocytes were lower in carcinoma tissue as compared to unaffected tissues. However, this difference was not statistically significant for the CD4^+^ T lymphocytes (Figs. 3a, d, 4c). An apparent trend towards lower chemokine receptor expression by T lymphocytes from carcinoma tissue was observed for the receptors CCR3 and CX3CR1, whereas CXCR5 was not expressed at all by T lymphocytes from carcinoma tissue (Figs. 3a–f, 4b, d). Thus, although the general pattern of chemokine receptor expression by T lymphocytes in carcinoma tissue as compared with unaffected mucosa was quite similar, there were clear and statistically significant differences with regard to a number of chemokine receptors, i.e., CCR2 and CXCR5 in CD4^+^ T lymphocytes, and CCR2, CCR9, and CXCR5 in CD8^+^ T lymphocytes.

## Discussion

High numbers of human tumor-infiltrating T lymphocytes (TILs) have been demonstrated to correlate with enhanced survival [27–29, 33]. Furthermore, increased frequencies of intratumoral CD4^+^ and CD8^+^ T lymphocytes have been independently associated with good clinical outcome [46]. Despite a clear increase in the total number of T lymphocytes in carcinoma tissue, it has been shown that the relative proportions of CD4^+^ and CD8^+^ T lymphocytes remain the same [30, 44]. In this study, we have examined the chemokine receptor expression on CD4^+^ and CD8^+^ T lymphocytes, respectively, isolated from CRC tissue, and compared this to CD4^+^ and CD8^+^ T lymphocytes isolated from nearby located unaffected colonic mucosal tissue.

The recruitment of T lymphocytes from the circulation into the tumor mass is a highly regulated process mediated by adhesion molecules, chemokines expressed by the tissue, and chemokine receptors expressed by the lymphocytes [33, 46]. In murine cancer models, agents blocking chemokines or their receptors have been shown to inhibit tumor growth, and moreover, this effect correlated with TILs [47–49]. However, the role of different chemokine receptors in the recruitment of human T lymphocytes to tumor tissues has not been fully elucidated.

In this study, we examined the expression of 16 different chemokine receptors on CD4^+^ and CD8^+^ T lymphocytes, respectively. This represents a notably broad panel in comparison with previously published studies, both in the perspective of normal colonic tissue and CRC tissue [9, 44]. The results showed that the isolated T lymphocytes expressed all the examined chemokine receptors, to various degrees, with the exception of CCR8, CCR10, CXCR1, and CXCR2. This is in agreement with a meticulous study examining the expression of 11 different chemokine receptors on peripheral blood lymphocytes [50]. It has been suggested by other groups that T helper subsets (i.e., Th1, Th2, and Th17) [51–53] and cytotoxic T lymphocyte subsets (i.e., Tc1 and Tc2) [54] have specific chemokine receptor expression patterns, but subsequent studies have shown that the overlap between the subpopulations is substantial and that the chemokine receptor profile can change without a concomitant change in the cytokine profile [50, 55–58]. Thus, the use of chemokine receptors as markers for T lymphocyte subsets is controversial. In our study, CCR5, CXCR3, and CXCR4 were the receptors displaying the highest expression frequencies, which interestingly enough was true for both CD4^+^ and CD8^+^ T lymphocytes. Although the chemokine receptor expression was in large parts similar between CD4^+^ and CD8^+^ T lymphocytes from unaffected mucosa, a number of the receptors displayed significant differences in expression. Specifically, CCR2 and CCR4 were more frequently expressed by CD4^+^ T lymphocytes, whereas CXCR3 and CXCR6 were expressed at higher levels by CD8^+^ T lymphocytes. These results suggest that the molecular mechanisms mediating T lymphocyte recruitment into the gut mucosa may differ between CD4^+^ and CD8^+^ T lymphocytes, as illustrated by significant differences in the levels of one-third of the expressed chemokine receptors.

The results from this study showed that the expression levels of certain chemokine receptors differed significantly when T lymphocytes isolated from colonic carcinoma tissue were matched against those isolated from unaffected colonic mucosal tissue. The proportion of CCR2^+^ cells was around 50% lower among both CD4^+^ and CD8^+^ T lymphocytes in tumor as compared with unaffected tissue. Interestingly, CCR2 has been associated with Th1, Th17, and Tc1 but not with other T lymphocyte subsets [46, 54, 59, 60]. Thus, the lower frequency of CCR2^+^ T lymphocytes in carcinoma tissues might be a result of tumor immune surveillance escaping mechanisms [46]. Intriguingly, it has been described that tumors may alter the chemistry of certain chemokines by nitrosylation and thereby hindering T lymphocyte infiltration [61]. Indeed, tumor-produced nitrosylated CCL2, which is the main ligand for CCR2 [62], has been shown to selectively recruit myeloid-derived suppressor cells (MDSCs) while abrogating recruitment of Th1 and cytotoxic T lymphocytes [46, 61, 63].

Svensson et al. analyzed seven different chemokine receptors in CRC patients in a similar fashion as we did and reported that the percentage of CXCR3^+^ cells among CD4^+^ and CD8^+^ T lymphocytes was lower in colorectal carcinoma tissue as compared with unaffected tissue, the percentage of CCR4^+^ cells was higher among tumor CD4^+^ but not CD8^+^ T lymphocytes, whereas five other chemokine receptors did not show any differences [44]. Our data showed a numerical, but not statistically significant decrease, in tumor CXCR3^+^ CD4^+^ and CD8^+^ T lymphocytes, whereas CCR4^+^ CD4^+^ and CD8^+^ T lymphocytes were present at equal frequencies in tumor and in unaffected tissue. However, while their analysis of CCR9 did not show any differences, our results showed a significant decrease in the percentage of CCR9^+^ among CD8^+^ T lymphocytes in tumor compared to unaffected tissue, and CCR9^+^CD4^+^ T lymphocytes were numerically but not significantly fewer in CRC tissue. It is well established that CCR9 mediates gut-specific homing of T lymphocytes and that gut epithelial cells produce CCL25 (CCR9 ligand) [64–66]. Remarkably, Chen et al showed that CCR9 was upregulated in intestinal adenomas, colonic carcinoma in situ, primary CRC cell cultures, and colon-cancer-initiating cell (CCIC) lines [67]. The authors suggested that locally produced CCL25 by the surrounding colon epithelium binds to the epithelial-expressed CCR9 and promotes proliferation of the early stage CRC cells. In addition, they describe a CCL25 “sink” function for epithelial-expressed CCR9 [67, 68]. Thus, a functional down-regulation of CCL25-mediated T lymphocyte recruitment in CRC tissue could potentially explain our results showing decreased numbers of CCR9^+^ T lymphocytes in carcinoma tissues compared to unaffected tissues. In addition, this could represent a novel immune surveillance evasion mechanism used by early stage CRC.

Interestingly, we did not detect any CXCR5^+^ T lymphocytes in the carcinoma tissues, in contrast to the unaffected tissues. Although there have been reports on increased expression of CXCL13 (the only known ligand for CXCR5) in CRC, our data are in agreement with the study by Bindea et al. describing an immune evasive mechanism in CRC through which tumor CXCL13 production is downregulated (chromosomal deletion) and CXCR5^+^ lymphocytes hindered from being recruited to the tumor [69–71].

## Conclusions

Using a broad antibody panel to 16 different chemokine receptors, our study showed that T lymphocytes which had infiltrated into colorectal carcinoma tissue differed significantly in their expression of CCR2, CCR9, and CXCR5, thus displaying a distinct chemokine receptor profile as compared with T lymphocytes in unaffected colonic mucosa. The specific differences in CCR2 and CXCR5 expression fit well with two previously described immune surveillance evasion mechanisms, respectively, used by cancers [61, 69]. In addition, we suggest a novel potential immune surveillance evasion mechanism through which colorectal carcinoma tissue perturbs the ability of CCR9^+^ T lymphocytes to be recruited to the tumor. Hence, manipulation of chemokine/chemokine receptor axes’ biology by CRC seems to represent an important mechanism limiting the anti-tumor immune response. Finally, the expression profile differed in one-third of the expressed chemokine receptors between CD4^+^ and CD8^+^ T lymphocytes in normal colonic mucosa. These data contribute to the understanding of how T lymphocyte trafficking to the human colon mucosa is regulated under normal conditions, an area that remains surprisingly ambiguous. Taken together, this study should be followed by larger confirming studies which will have great potential to lay the basis for the development of new cancer immunotherapeutics. Such agents should exploit trafficking mechanisms of T lymphocytes and aim at changing the tumor microenvironment, so that it becomes more extensively infiltrated by T lymphocytes. Gratifyingly, the initial studies along that path trying to increase the levels of cancer-related pro-inflammatory danger signals and intratumoral cytokines have already been performed [72, 73].

## Abbreviations

CRC: colorectal cancer
CTLA-4: cytotoxic T lymphocyte-associated protein-4
IEL: intraepithelial lymphocytes
IQR: interquartile range
LP: lamina propria
Tc: cytotoxic T lymphocyte
Th: helper T lymphocyte
TIL: tumor-infiltrating T lymphocytes
7AAD: 7-amino-actinomycin D

## References

[1] Boyle P, Ferlay J (2005). Cancer incidence and mortality in Europe, 2004. Ann Oncol.

[2] de la Cruz-Merino L, Grande-Pulido E, Albero-Tamarit A, de Villena MEC-M (2008). Cancer and immune response: old and new evidence for future challenges. Oncologist.

[3] Golby SJ, Chinyama C, Spencer J (2002). Proliferation of T-cell subsets that contact tumour cells in colorectal cancer. Clin Exp Immunol.

[4] Nomiyama H, Osada N, Yoshie O (2013). Systematic classification of vertebrate chemokines based on conserved synteny and evolutionary history. Genes Cells.

[5] Zlotnik A, Yoshie O (2012). The chemokine superfamily revisited. Immunity.

[6] Moser B, Wolf M, Walz A, Loetscher P (2004). Chemokines: multiple levels of leukocyte migration control. Trends Immunol.

[7] Levoye A, Dam J, Ayoub MA, Guillaume JL, Jockers R (2006). Do orphan G-protein-coupled receptors have ligand-independent functions? New insights from receptor heterodimers. EMBO Rep.

[8] Luster AD (1998). Chemokines–chemotactic cytokines that mediate inflammation. N Engl J Med.

[9] Agace WW, Roberts AI, Wu L, Greineder C, Ebert EC, Parker CM (2000). Human intestinal lamina propria and intraepithelial lymphocytes express receptors specific for chemokines induced by inflammation. Eur J Immunol.

[10] Raz E, Mahabaleshwar H (2009). Chemokine signaling in embryonic cell migration: a fisheye view. Development.

[11] Balkwill FR (2012). The chemokine system and cancer. J Pathol.

[12] Lazennec G, Richmond A (2010). Chemokines and chemokine receptors: new insights into cancer-related inflammation. Trends Mol Med.

[13] Murphy PM (2001). Chemokines and the molecular basis of cancer metastasis. N Engl J Med.

[14] Jones S, Chen WD, Parmigiani G (2008). Comparative lesion sequencing provides insights into tumor evolution. Proc Natl Acad Sci USA.

[15] Bozic I, Antal T, Ohtsuki H (2010). Accumulation of driver and passenger mutations during tumor progression. Proc Natl Acad Sci USA.

[16] Guy-Grand D, DiSanto JP, Henchoz P, Malassis-Seris M, Vassalli P (1998). Small bowel enteropathy: role of intraepithelial lymphocytes and of cytokines (IL-12, IFN-gamma, TNF) in the induction of epithelial cell death and renewal. Eur J Immunol.

[17] Komano H, Fujiura Y, Kawaguchi M (1995). Homeostatic regulation of intestinal epithelia by intraepithelial gamma delta T cells. Proc Natl Acad Sci USA.

[18] Norazmi MN, Hohmann AW, Skinner JM, Jarvis LR, Bradley J (1990). Density and phenotype of tumour-associated mononuclear cells in colonic carcinomas determined by computer-assisted video image analysis. Immunology.

[19] Hesterberg PE, Winsor-Hines D, Briskin MJ (1996). Rapid resolution of chronic colitis in the cotton-top tamarin with an antibody to a gut-homing integrin alpha 4 beta 7. Gastroenterology.

[20] Hosoe N, Miura S, Watanabe C (2004). Demonstration of functional role of TECK/CCL25 in T lymphocyte–endothelium interaction in inflamed and uninflamed intestinal mucosa. Am J Physiol Gastrointest Liver Physiol.

[21] Wang C, Hanly EK, Wheeler LW, Kaur M, McDonald KG, Newberry RD (2010). Effect of alpha4beta7 blockade on intestinal lymphocyte subsets and lymphoid tissue development. Inflamm Bowel Dis.

[22] Connor SJ, Paraskevopoulos N, Newman R (2004). CCR2 expressing CD4+ T lymphocytes are preferentially recruited to the ileum in Crohn’s disease. Gut.

[23] Diegelmann J, Seiderer J, Niess JH (2010). Expression and regulation of the chemokine CXCL16 in Crohn’s disease and models of intestinal inflammation. Inflamm Bowel Dis.

[24] Kunkel EJ, Boisvert J, Murphy K (2002). Expression of the chemokine receptors CCR4, CCR5, and CXCR3 by human tissue-infiltrating lymphocytes. Am J Pathol.

[25] Papadakis KA, Prehn J, Zhu D (2004). Expression and regulation of the chemokine receptor CXCR3 on lymphocytes from normal and inflammatory bowel disease mucosa. Inflamm Bowel Dis.

[26] Yuan YH, ten Hove T, The FO, Slors JF, van Deventer SJ, te Velde AA (2001). Chemokine receptor CXCR3 expression in inflammatory bowel disease. Inflamm Bowel Dis.

[27] Pages F, Berger A, Camus M (2005). Effector memory T cells, early metastasis, and survival in colorectal cancer. N Engl J Med.

[28] Pages F, Kirilovsky A, Mlecnik B (2009). In situ cytotoxic and memory T cells predict outcome in patients with early-stage colorectal cancer. J Clin Oncol.

[29] Galon J, Costes A, Sanchez-Cabo F (2006). Type, density, and location of immune cells within human colorectal tumors predict clinical outcome. Science.

[30] Fridman WH, Pages F, Sautes-Fridman C, Galon J (2012). The immune contexture in human tumours: impact on clinical outcome. Nat Rev Cancer.

[31] Onizuka S, Tawara I, Shimizu J, Sakaguchi S, Fujita T, Nakayama E (1999). Tumor rejection by in vivo administration of anti-CD25 (interleukin-2 receptor alpha) monoclonal antibody. Cancer Res.

[32] Yu P, Fu YX (2006). Tumor-infiltrating T lymphocytes: friends or foes?. Lab Investig.

[33] Oelkrug C, Ramage JM (2014). Enhancement of T cell recruitment and infiltration into tumours. Clin Exp Immunol.

[34] Jass JR (1986). Lymphocytic infiltration and survival in rectal cancer. J Clin Pathol.

[35] Svennevig JL, Lunde OC, Holter J, Bjorgsvik D (1984). Lymphoid infiltration and prognosis in colorectal carcinoma. Br J Cancer.

[36] Baier PK, Eggstein S, Wolff-Vorbeck G, Baumgartner U, Hopt UT (2005). Chemokines in human colorectal carcinoma. Anticancer Res.

[37] Erreni M, Bianchi P, Laghi L (2009). Expression of chemokines and chemokine receptors in human colon cancer. Methods Enzymol.

[38] Correale P, Rotundo MS, Botta C (2012). Tumor infiltration by T lymphocytes expressing chemokine receptor 7 (CCR7) is predictive of favorable outcome in patients with advanced colorectal carcinoma. Clin Cancer Res.

[39] Correale P, Rotundo MS, Botta C, Del Vecchio MT, Tassone P, Tagliaferri P (2012). Tumor infiltration by chemokine receptor 7 (CCR7)(+) T-lymphocytes is a favorable prognostic factor in metastatic colorectal cancer. Oncoimmunology.

[40] Cozar JM, Canton J, Tallada M (2005). Analysis of NK cells and chemokine receptors in tumor infiltrating CD4 T lymphocytes in human renal carcinomas. Cancer Immunol Immunother.

[41] Liu J, Zhang N, Li Q (2011). Tumor-associated macrophages recruit CCR6+ regulatory T cells and promote the development of colorectal cancer via enhancing CCL20 production in mice. PLoS ONE.

[42] Rani AS, Saritha K, Nagamani V, Sulakshana G (2011). In vitro evaluation of antifungal activity of the seed extract of *Embelia ribes*. Indian J Pharm Sci.

[43] Vicari AP, Ait-Yahia S, Chemin K, Mueller A, Zlotnik A, Caux C (2000). Antitumor effects of the mouse chemokine 6Ckine/SLC through angiostatic and immunological mechanisms. J Immunol.

[44] Svensson H, Olofsson V, Lundin S (2012). Accumulation of CCR4(+)CTLA-4 FOXP3(+)CD25(hi) regulatory T cells in colon adenocarcinomas correlate to reduced activation of conventional T cells. PLoS ONE.

[45] Fiocchi C, Youngman KR. Isolation of human intestinal mucosal mononuclear cells. Curr Protoc Immunol. 2001;Chapter 7:Unit 7.30. 10.1002/0471142735.im0730s19 18432842

[46] Melero I, Rouzaut A, Motz GT, Coukos G (2014). T-cell and NK-cell infiltration into solid tumors: a key limiting factor for efficacious cancer immunotherapy. Cancer Discov.

[47] Braun SE, Chen K, Foster RG (2000). The CC chemokine CK beta-11/MIP-3 beta/ELC/Exodus 3 mediates tumor rejection of murine breast cancer cells through NK cells. J Immunol.

[48] Fushimi T, O’Connor TP, Crystal RG (2006). Adenoviral gene transfer of stromal cell-derived factor-1 to murine tumors induces the accumulation of dendritic cells and suppresses tumor growth. Cancer Res.

[49] Sharma S, Stolina M, Luo J (2000). Secondary lymphoid tissue chemokine mediates T cell-dependent antitumor responses in vivo. J Immunol.

[50] Kim CH, Rott L, Kunkel EJ (2001). Rules of chemokine receptor association with T cell polarization in vivo. J Clin Investig.

[51] Acosta-Rodriguez EV, Rivino L, Geginat J (2007). Surface phenotype and antigenic specificity of human interleukin 17-producing T helper memory cells. Nat Immunol.

[52] Sallusto F, Lenig D, Mackay CR, Lanzavecchia A (1998). Flexible programs of chemokine receptor expression on human polarized T helper 1 and 2 lymphocytes. J Exp Med.

[53] Sallusto F, Mackay CR, Lanzavecchia A (1997). Selective expression of the eotaxin receptor CCR3 by human T helper 2 cells. Science.

[54] Cerwenka A, Morgan TM, Harmsen AG, Dutton RW (1999). Migration kinetics and final destination of type 1 and type 2 CD8 effector cells predict protection against pulmonary virus infection. J Exp Med.

[55] Aarvak T, Strand E, Teigland J, Miossec P, Natvig JB (2001). Switch in chemokine receptor phenotype on memory T cells without a change in the cytokine phenotype. Scand J Immunol.

[56] Holse M, Assing K, Poulsen LK (2006). CCR3, CCR5, CCR8 and CXCR3 expression in memory T helper cells from allergic rhinitis patients, asymptomatically sensitized and healthy individuals. Clin Mol Allergy.

[57] Nanki T, Lipsky PE (2000). Lack of correlation between chemokine receptor and T(h)1/T(h)2 cytokine expression by individual memory T cells. Int Immunol.

[58] Viola A, Luster AD (2008). Chemokines and their receptors: drug targets in immunity and inflammation. Annu Rev Pharmacol Toxicol.

[59] Ward SG, Marelli-Berg FM (2009). Mechanisms of chemokine and antigen-dependent T-lymphocyte navigation. Biochem J.

[60] Zhang HH, Song K, Rabin RL (2010). CCR2 identifies a stable population of human effector memory CD4+ T cells equipped for rapid recall response. J Immunol.

[61] Molon B, Ugel S, Del Pozzo F (2011). Chemokine nitration prevents intratumoral infiltration of antigen-specific T cells. J Exp Med.

[62] Ling MF, Luster AD (2011). Novel approach to inhibiting chemokine function. EMBO Mol Med.

[63] Chun E, Lavoie S, Michaud M (2015). CCL2 promotes colorectal carcinogenesis by enhancing polymorphonuclear myeloid-derived suppressor cell population and function. Cell Rep.

[64] Marsal J, Agace WW (2012). Targeting T-cell migration in inflammatory bowel disease. J Intern Med.

[65] Svensson M, Marsal J, Ericsson A (2002). CCL25 mediates the localization of recently activated CD8alphabeta(+) lymphocytes to the small-intestinal mucosa. J Clin Investig.

[66] Zabel BA, Agace WW, Campbell JJ (1999). Human G protein-coupled receptor GPR-9-6/CC chemokine receptor 9 is selectively expressed on intestinal homing T lymphocytes, mucosal lymphocytes, and thymocytes and is required for thymus-expressed chemokine-mediated chemotaxis. J Exp Med.

[67] Chen HJ, Edwards R, Tucci S (2012). Chemokine 25-induced signaling suppresses colon cancer invasion and metastasis. J Clin Investig.

[68] Ulvmar MH, Hub E, Rot A (2011). Atypical chemokine receptors. Exp Cell Res.

[69] Bindea G, Mlecnik B, Tosolini M (2013). Spatiotemporal dynamics of intratumoral immune cells reveal the immune landscape in human cancer. Immunity.

[70] Qi XW, Xia SH, Yin Y (2014). Expression features of CXCR5 and its ligand, CXCL13 associated with poor prognosis of advanced colorectal cancer. Eur Rev Med Pharmacol Sci.

[71] Zhu Z, Zhang X, Guo H, Fu L, Pan G, Sun Y (2015). CXCL13-CXCR5 axis promotes the growth and invasion of colon cancer cells via PI3K/AKT pathway. Mol Cell Biochem.

[72] Brody JD, Ai WZ, Czerwinski DK (2010). In situ vaccination with a TLR9 agonist induces systemic lymphoma regression: a phase I/II study. J Clin Oncol.

[73] Kroemer G, Galluzzi L, Kepp O, Zitvogel L (2013). Immunogenic cell death in cancer therapy. Annu Rev Immunol.

